# Laser-Induced Interference to Infrared Detector Using Continuous Wave and Short-Pulse Lasers

**DOI:** 10.3390/s24154885

**Published:** 2024-07-27

**Authors:** Yingjie Ma, Weijing Zhou, Hao Chang, Zhilong Jian

**Affiliations:** Department of Aerospace and Technology, Space Engineering University, Beijing 101416, China; mayingjie@hgd.edu.cn (Y.M.); changhao5976911@163.com (H.C.); jzl862366@163.com (Z.J.)

**Keywords:** CW laser, pulsed laser, PbS infrared detector, interference threshold, interference testing

## Abstract

The response of a DPbS3200 infrared detector irradiated by a nanosecond pulsed laser and CW laser has been investigated to study laser-induced interference. A laser interference experiment system was constructed to measure the time-varying response signal. A nanosecond pulsed laser and a CW laser of 10 Hz were used, with a 1064 nm wavelength and a millimeter-scale irradiation spot diameter. Firstly, the characteristics of transient interference signals induced by pulsed lasers were analyzed. Then, the characteristics of response signal interference by both CW laser and pulsed laser irradiation were further investigated. The results showed that the pulsed laser only produced transient interference. However, the CW laser led to a significant amplitude reduction of the response signal, which could continuously interfere in the operating time. For transient interferences, the amplitude of the interference signal increased linearly with the laser fluence. The relation between the pulse repetition rate of the incident laser and the operating frequency of the detector determined the numbers of transient interference signals in one response period; for the interference induced by both the CW laser and pulsed laser, CW laser interference played a leading role when CW laser power density increased to 4.1 W/cm^2^ or more. As the CW laser fluence reached 6.1 W/cm^2^, the PbS infrared detector was no longer able to detect any signal, which caused temporary blindness. In the end, a probit model was used to determine the interference threshold.

## 1. Introduction

IR radiation is widely used in environmental monitoring, non-contact detecting, aerospace, and other fields [1,2,3]. Compared with normal CCD cameras, infrared detectors have many advantages such as high quantum efficiency, a large absorption coefficient, a wide response wave band, as well as better detectivity [4,5]. The function of the infrared detector is to convert an incident infrared radiation signal into an electrical signal output [6,7]. However, as a weak optical type of detector, it has a large optical gain. This leads to the fact that infrared detectors are extremely easy to be interfered with or even damaged by a high-power laser, which is one of the most common factors affecting normal operation, as well as lifetime [8,9,10]. Up until now, investigations into the characteristics of the laser-induced interference signal of infrared detectors with different paraments are still insufficient. Therefore, it deserves more attention due to its value in optimizing and improving detector design, enhancing performance and lifetime, and preventing interference from occurring.

Experimental investigations, as well as simulations, have been conducted in the field of laser-induced infrared detector failure. In an early systemic study, F. Bartoli et al. investigated the thermal damage effects of 1.06 μm-, 5.2 μm-, and 10.6 μm-band pulsed lasers on typical infrared materials, respectively [11]. The thermal damage thresholds of InSb, HgGeTe, polycrystalline Si, and PbS were experimentally compared. In 2021, N. I. Pavlov et al. studied the effect of out-of-field laser interference on the detection capability of an infrared optoelectronic system (IROES) model [12]. It was shown that the intensity distribution of scattered laser radiation formed in the matrix photodetector (MPD) plane has a speckle character, and laser interference generates a noisy signal that reduces the detection capability of the IROES. In 2023, Wang et al. investigated the interference of an HgCdTe infrared focal plane array (IRFPA) detector that was irradiated by a high-repetition-rate, mid-infrared CW laser [13]. It was shown that the performance of the detector is extremely sensitive to laser power, with a temporary blindness threshold of 0.14 W/cm^2^ in the case of multi-pulse accumulation.

As a typical shortwave infrared detection material, PbS has a more satisfactory response in the shortwave infrared band, which is one of the atmospheric windows with the highest transmittance [14]. Therefore, PbS infrared detectors are widely used in astronomical observation, aerospace, meteorological monitoring, and other fields [15,16]. Yuan et al. conducted experiments on the irradiation effects of a continuous oxygen–iodine laser on a Ge-PbS detector [17]. A blinding interference occurred when the laser power was 17 W, and a permanent failure happened when the laser power was 198W with a spot diameter of 2.1 mm. In 2022, Wang et al. carried out damage experiments of mid-infrared lasers with different pulse repetition rates on a PbS detector [18]. The results showed that the damage threshold of a laser with a wavelength of 2.79 μm is 13.03 J/cm^2^, and the pulse number, as well as the pulse repetition rate, have significant influence on the damage effect. Numerical simulations of the temperature distribution and splashing particle trajectory of the PbS detector were also performed. Nevertheless, few experimental investigations about the nanosecond laser interference of PbS detectors have been conducted in related fields, including the results of the irradiation of both CW lasers and pulsed lasers.

To make up for this gap, this study aimed to determine the interference effects of a PbS infrared detector induced by nanosecond pulsed and CW lasers. A laser interference experimental system was constructed to measure the time-varying response signal of the detector accurately. The differences in the interference signals induced by pulsed lasers and CW lasers were discussed. For pulsed-laser irradiation, the interference signal manifested itself as a step change in the time-varying response signal. In the case of CW laser irradiation, the interference was mainly characterized by a decrease in the amplitude of the response signal accompanied by fluctuations. Based on this, both the amplitude and frequency influences of the interference signals were further analyzed. In the end, the rules of the interference induced by both the CW laser and pulsed laser were summarized.

## 2. Experimental Set-Up

**Figure 1:**
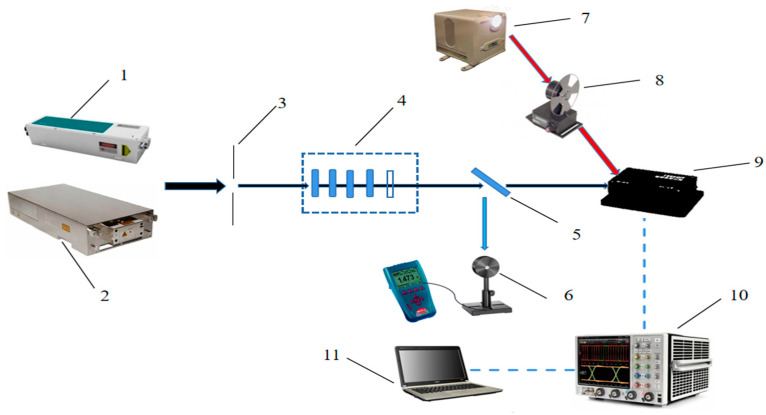
Schematic diagram of the experimental system of laser irradiation infrared detector. (1) Q-switched Nd:YAG laser; (2) PSU-H-LED model CW laser; (3) diaphragm; (4) attenuator group; (5) beam splitter mirror; (6) laser energy detector; (7) CW white light source; (8) chopper; (9) PbS infrared detector; (10) oscilloscope; (11) computer. The blue arrows indicate the optical circuit of the laser damage infrared detector. The red arrows indicate the optical circuit of the normal operating signal. The blue dash lines represent the measurement of the time-varying response signal.

The schematic diagram of the experimental system is shown in Figure 1. The laser pulse with a central wavelength of 1064 nm and 10 ns full-width at half maximum (FWHM) was emitted by a Q-switched Nd:YAG laser with a maximum pulse energy of 900 mJ. A CW (model PSU-H-LED) laser was used to emit the 1064 nm CW laser with a peak power of 1 W. The laser passed through the attenuator group and then irradiated the light-sensitive element of the PbS infrared detector. A white light source, as well as a chopper, were employed to let the detector produce sine-like signals with different frequencies. During the experiment, the sine-like signals were regarded as the normal response signal of the detector. An oscilloscope (1.25 GSa/s sampling rate, 50 Mpts maximum memory depth) was used to record the time-varying response signal of the PbS infrared detector. Laser interference would lead to the fluctuations and step changes of the time-varying response signal. The diameter of the irradiated spot was measured by a beam quality analyzer. A beam splitter directed a proportion of the beam to a calibrated reference energy detector (model Ophir PD10C, Ophir, Jerusalem, Israel). The incident laser energy was calculated in real time through splitting ratios. In the experiment, the interference signal could be observed when the pulsed laser fluence reached 2.0 J/cm^2^. Therefore, this fluence was chosen as the lowest reference value.

The PbS (model DPbS3200, Zolix, Beijing, China) infrared detector with a pre-amplifier and amplification circuit was selected as the interference target. Though different amplification circuits would produce changes in the results, in our investigation, we only focused on a specific amplification circuit. The parameters of the detector used in this experiment are presented in Table 1. The PbS detector is a kind of lead salt infrared photoelectric effect sensor with a primary response wavelength of shortwave infrared (1.0–3.0 μm). Based on the photoconductive effect of the semiconductor materials, hole electron pairs are generated between the conduction and valence bands through the irradiation of incident photons [19]. When a photoelectron meets electron hole pairs, a charge separation will occur, resulting in the electric current, which converts the infrared radiation into the electrical signal [20,21,22].

When the laser irradiated the light-sensitive material of the detector, the surface irradiation zone was quickly heated and formed optical modulation and stress. The coupling of these effects would result in degradation of the IR detector response, including, but not limited to noise growth; amplitude change; and signal fluctuation. This kind of temporary degradation lasted until the irradiation ended, which was defined as interference. The oscilloscope was used to measure the changes of the time-varying interference signal, so as to quantitatively evaluate the interference effect. Five groups of repeated experiments were performed for each laser parameter to reduce experimental errors. The average value and error of the signal amplitude, as well as its attenuation rate, were calculated.

## 3. Results and Discussion

### 3.1. Transient Interference Induced by Nanosecond Pulsed Laser

Prior to the experiment, the surface of the optical lens was wiped with alcohol in order to prevent the interference effect from being affected by impurities on the surface of the optical lens. The beam size was approximately 500 μm at 1/e^2^, and the laser pulse duration was 10 ns. During the experiment, the laser energy was gradually increased. The range of pulsed laser energy was 4.0~24.0 mJ, corresponding to a laser fluence of 2.0~12.3 J/cm^2^.

**Figure 2:**
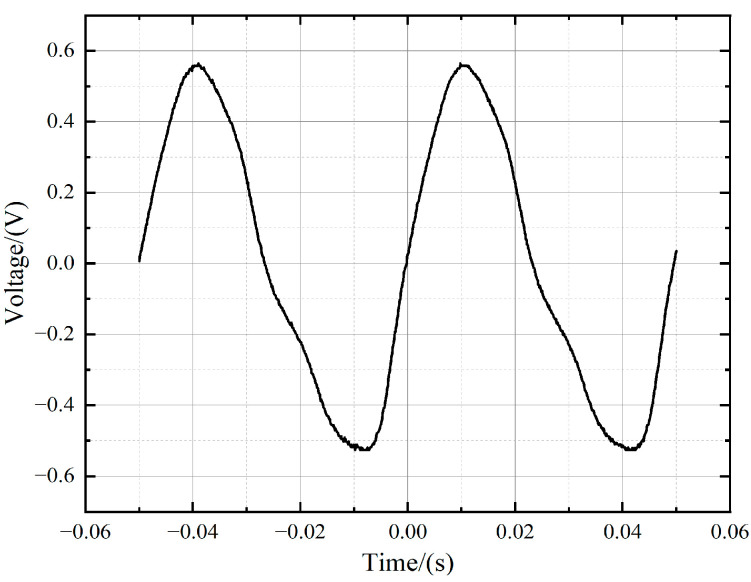
The normal operation signal of PbS detector by CW white light and chopper.

**Figure 3:**
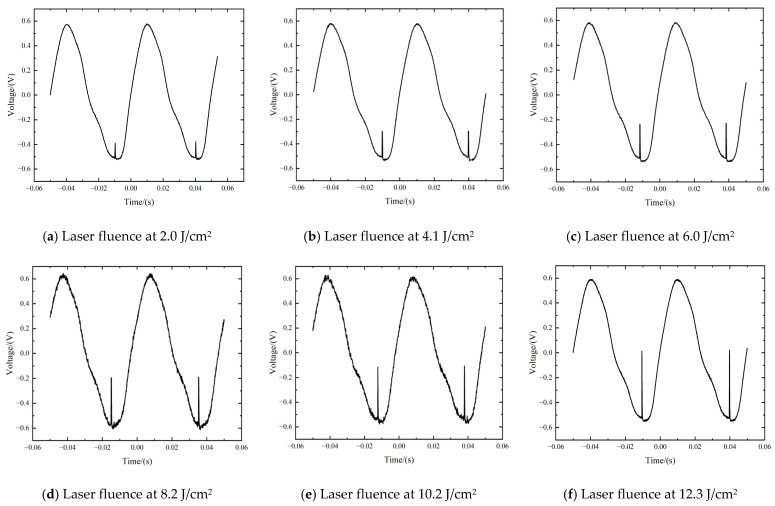
Time-varying response signal of PbS detector with different pulsed laser fluence.

**Figure 4:**
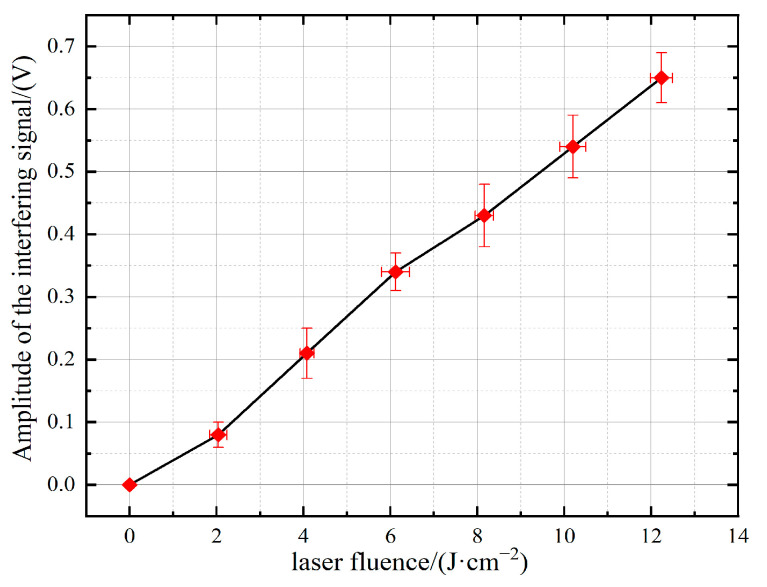
The relationship between pulsed laser fluence and transient interference signal amplitude.

Before laser irradiation, the PbS detector generated the sine-like response signal due to the CW white light source and the chopper, which is presented in Figure 2. However, with the irradiation of pulsed lasers, as shown in Figure 3, transient interferences happened. The duration of a single transient interference signal approximated the pulse-width time of the laser. Interference manifested itself as a step change in the time-varying response signal. A measurable interference signal corresponding to a laser fluence threshold of 2.0 J/cm^2^ is presented in Figure 3a. As shown in Figure 3f, when the laser fluence reached 12.3 J/cm^2^ or more, the peak value of the transient interference signal increased to 0.6 V, which was above the amplitude of the initial sine-like detection signal. The relationship between the pulsed laser fluence and the interference signal amplitude was further investigated, which is shown in Figure 4. It can be seen that the interference signal amplitude increased linearly with the growth of the laser fluence. The larger the pulsed laser fluence, the greater the amplitude of the interference signal, leading to a more significant transient interference effect.

**Figure 5:**
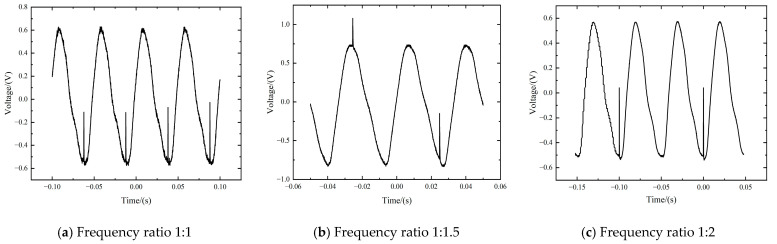
Time-varying response signals with different pulse repetition rate ratios between the chopper and the multi-pulse laser.

Pulsed lasers with different pulse repetition rates were used as the interference source in order to investigate the frequency influence of the signal. During the experiment, the laser fluence was set to the value of 8.2 J/cm^2^. The pulse repetition rate of the pulsed laser and chopper was adjusted to 20 Hz + 20 Hz, 20 Hz + 30 Hz, as well as 10 Hz + 20 Hz, respectively, corresponding to the frequency ratios of 1:1, 1:1.5, and 1:2. Figure 5 presents the time-varying response signals of PbS detectors with different pulse repetition rate ratios. As shown in Figure 5a, when the frequency ratio was 1:1, each period only presented one transient interference signal at the wave trough of the sine-like detector signal; in Figure 5b, when the frequency ratio reached 1:1.5, interfering signals alternately appeared on the peak or trough of the signal. As for Figure 5c, only one interference signal was formed within two periods of the sine-like signal. It can be deduced that the relation between the pulse repetition rate of the incident laser and the operating frequency of the detector determines whether the multi-pulse laser can interfere within one signal period or not. When the laser irradiation frequency is larger than or equal to the operating frequency of the detector, at least one transient interference is ensured for each signal period. However, as the laser pulse-repetition rate is the lesser one, transient interference signals need more periods to occur. Therefore, the incidence of lasers with high pulse-repetition rates on infrared detectors should be strictly avoided.

### 3.2. Sustained Interference Induced by Both Pulsed Laser and CW Laser

**Figure 6:**
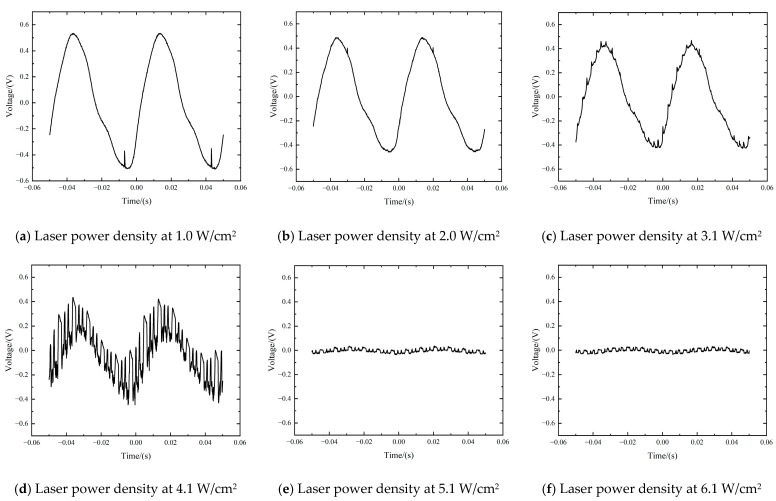
Time-varying response signals with the irradiation of both pulsed laser and CW laser of various power density. (Pulsed laser fluence is set to 8.2 J/cm^2^).

Both the pulsed laser and CW laser were used to study the results of irradiation of both the CW laser and pulsed laser. The laser irradiation duration was 0.8 s. During the experiment, the laser fluence of the pulsed laser was set to 8.2 J/cm^2^ constantly, but the CW laser energy was gradually increased. The range of the CW laser power was 2.0~12.0 mW, corresponding to a laser power density of 1.0~6.1 W/cm^2^. Time-varying response signals by the CW laser and pulsed laser are presented in Figure 6. It can be seen that the pulsed laser only produced transient interference to the infrared detector. However, the CW laser can lead to continuous interference signals, which result in the failure of the detector for a long time. In Figure 6a–c, when the CW laser power density was in the range of 1.0~2.0 W/cm^2^, the irradiation of the CW laser mainly reduced the amplitude of the pulsed laser transient interference signal. A significant fluctuation of the time-varying response signal can be clearly observed in Figure 6d as the CW laser power density reaches above 3.0 W/cm^2^. With the increase of the CW laser fluence, the response signal amplitude of the PbS detector became smaller and smaller. As shown in Figure 6e, when the laser power density was above 5.1 W/cm^2^, the signal became undetectable. As the CW laser fluence increased further, the time-varying response signal tended to curve with no signal output. In this process, optical effects gradually began to play a dominant role. Due to the optical saturation, the output signal eventually became undetectable.

**Figure 7:**
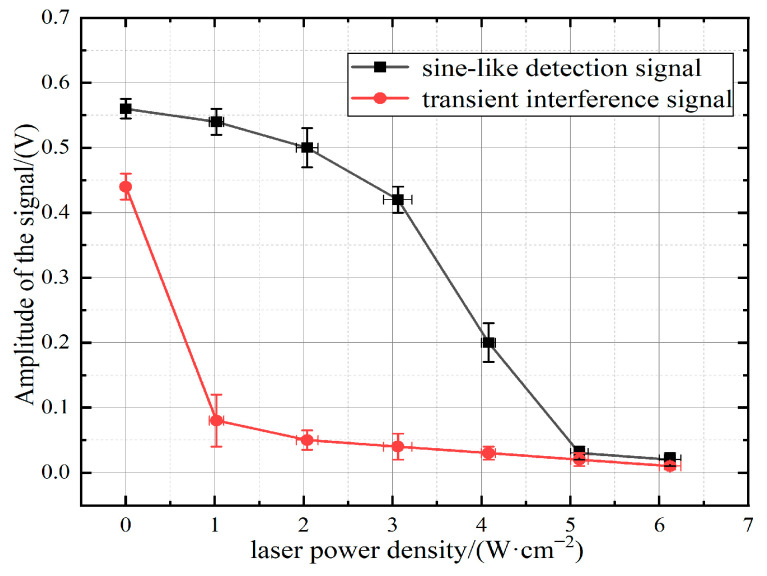
Amplitude changes of detector signal and pulsed laser interference signal.

As presented in Figure 7, when the CW laser power density was below 2.0 W/cm^2^, the normal response signal was almost free of interference, but the transient interference signal was attenuated sharply. It can be seen in Table 2 that, compared with Figure 3d, the amplitude of the transient interference signal was reduced by nearly 70%, though the incident laser fluence of the pulsed laser was the same. However, the amplitude attenuation rate of the detector signal was only less than 4%. This means that, in this process, the attenuation of the transient pulsed interference signal was significantly larger than the normal detector signal. However, this phenomenon can only be observed when the power density of the CW laser is below 2.0 W/cm^2^. As the CW laser power density increases, both the detector and the interference signal degrade rapidly.

As the CW laser power density increased to 4.1 W/cm^2^, the interference of the CW laser started to play a leading role, compared with transient interference. Large amplitude noise appeared in the sine-like signal, resulting in a reduction in the signal amplitude of approximately 50%. When the laser power density reached 6.1 W/cm^2^ or more, the amplitude attenuation rate was above 95%. Both the interfering signals of the pulsed laser and the sine-like detector signal were no longer being detected. It can be concluded that the CW laser of 6.1 W/cm^2^ sufficiently interfered with the signal detection, which resulted in a sustained function failure of the PbS infrared detector.

**Figure 8:**
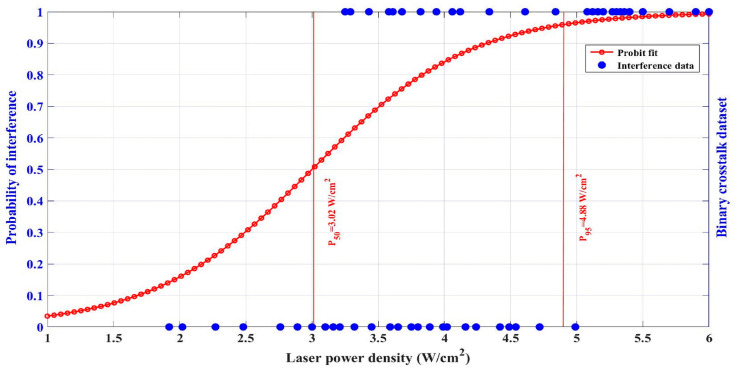
Interference probability of PbS detector induced by CW lasers of different power density.

It can be seen in Figure 6b–e that when the power density of the CW laser increased from 2.0 W/cm^2^ to 5.1 W/cm^2^, the response signal began to be disturbed severely, accompanied by a rapid decrease in the signal amplitude. Eventually, due to the optical saturation, the time-varying response signal became undetectable. Therefore, it is reasonable to assume that this power density range is a transitional phase of the complete interference. In Figure 6e,f, when no output signals are displayed, those are oscilloscope outputs without any sine-like signals; this is defined as “complete interference”. Otherwise, it is considered as “incomplete interference”. Based on the binary logistic regression [22,23], a probit model was used to determine the relationship between the probability of interference and the CW laser power density. To run the probit model, the data were first compressed into a binary dataset wherein results were categorized as a “complete interference” or “incomplete interference” event. For example, results that resulted in complete interference would be categorized as a “1”. Occurrences of incomplete interference would be categorized as “0”. Based on this, MathWorks MATLAB was used to run the regression on the binary dataset to generate the interference probability corresponding to different laser power densities. As shown in Figure 8, the interference probabilities of 50% and 95% corresponded to CW laser power densities of 3.0 W/cm^2^ and 4.9 W/cm^2^, respectively. Multiple sets of results showed that all probability points fell within the interference interval, as the power density was above 4.9 W/cm^2^. Thus, from the perspective of probability, we consider the laser power density of 4.9 W/cm^2^ can be used as the interference threshold of the CW laser.

## 4. Conclusions

In this study, the characteristics of the time-varying response signal of a PbS infrared detector irradiated by both a nanosecond pulsed laser and a CW laser were investigated. A laser interference experiment system was used to measure the time-varying interference signal and, thus, analyzed the degenerations of the signals induced by the lasers. The results show that the pulsed laser only interferes with the infrared detector transiently. It should be noted that the duration of each transient interference signal was about 400 μs. The CW laser of 10 Hz, however, induced sustained interference to the infrared detector, which can result in detector failure within the overall working time.

For pulsed laser interference, the performance of the PbS detector is sensitive to laser fluence. When the laser fluence gradually grows, the amplitude of the transient laser interference signal increases linearly. Pulsed laser irradiation does not change the frequency of the detection signal, but the pulse repetition rate of the laser can determine the number of transient interference signals. Both the fluence and the pulse repetition rate of the laser need consideration in detector protection.

As for the irradiation of both the CW laser and the pulsed laser, sustained amplitude degradations occur in the response signal. From the perspective of probability, the interference threshold of the CW laser is approximately 4.9 W/cm^2^. When the CW laser power density reaches 4.1 W/cm^2^, the interference effort of the CW laser starts to occupy a dominant role, and the signal gradually turns undetectable. With the laser power density increases, the frequency of the detection signal begins to change, and the amplitude attenuates sharply, resulting in the signal becoming gradually undetectable. As the CW laser power density reaches 6.1 W/cm^2^, the time-varying response signal tends to curve with extremely little change in amplitude. The PbS detector is no longer able to detect any signal, which must be avoided in engineering applications as much as possible. In a word, for DPbS3200 infrared detectors, continuous interference induced by CW lasers is obviously more severe than the transient interference of pulsed lasers. A pulsed laser of 12.3 J/cm^2^ and a CW laser of 4.1 W/cm^2^ can both result in signal distortion. A CW laser of 4.9 W/cm^2^ will totally lead to output signal failure.

## Figures and Tables

**Table 1:** The parameters of DPbS3200 PbS infrared detector used in this experiment.

Parameter	Value
Time constant (μs)	≤400
Peak wavelength (μm)	≥2.1
Frequency response range (Hz)	100–1000
Detection wavelength range (μm)	0.8–3.2
Magnification power	×1, ×10, ×100
Signal output mode	Voltage
Output signal polarity	+(P)

**Table 2:** The average amplitude attenuation rate of the detector signal and the pulsed laser interference signal under CW laser.

CW Laser Power Density (W/cm^2^)	Signal Amplitude Attenuation Rate (%)	Transient Interference Signal Attenuation Rate (%)
1.0	3.57	81.82
2.0	10.71	88.67
3.1	25.00	90.90
4.1	49.42	93.18
5.1	94.64	95.45
6.1	96.43	97.73

## Data Availability

The data presented in this study are available on request from the corresponding author. Informed consent was obtained from all subjects involved in the study.

## Nomenclature

CCD	Charge Coupled Device
CW	Continuous Wave
IROES	Infrared Optoelectronic System
MPD	Matrix Photodetector
IRFPA	Infrared Focal Plane Array
